# ERRFI1 exacerbates hepatic ischemia reperfusion injury by promoting hepatocyte apoptosis and ferroptosis in a GRB2-dependent manner

**DOI:** 10.1186/s10020-024-00837-4

**Published:** 2024-06-11

**Authors:** Hang Zhao, Huizi Mao

**Affiliations:** 1 Department of General Surgery, The First Affiliated Hospital of Jinzhou Medical University, Jinzhou, 121000 Liaoning China; 2 Department of Cardiology, The First Affiliated Hospital of Jinzhou Medical University, No. 2, Section 5, Renmin Street Guta District, Jinzhou, 121000 Liaoning China

**Keywords:** ERRFI1, Hepatic ischemia and reperfusion injury, Apoptosis, Ferroptosis, GRB2

## Abstract

**Background:**

Programmed cell death is an important mechanism for the development of hepatic ischemia and reperfusion (IR) injury, and multiple novel forms of programmed cell death are involved in the pathological process of hepatic IR. ERRFI1 is involved in the regulation of cell apoptosis in myocardial IR. However, the function of ERRFI1 in hepatic IR injury and its modulation of programmed cell death remain largely unknown.

**Methods:**

Here, we performed functional and molecular mechanism studies in hepatocyte-specific knockout mice and ERRFI1-silenced hepatocytes to investigate the significance of ERRFI1 in hepatic IR injury. The histological severity of livers, enzyme activities, hepatocyte apoptosis and ferroptosis were determined.

**Results:**

ERRFI1 expression increased in liver tissues from mice with IR injury and hepatocytes under oxygen-glucose deprivation/reoxygenation (OGD/R) conditions. Hepatocyte-specific ERRFI1 knockout alleviated IR-induced liver injury in mice by reducing cell apoptosis and ferroptosis. ERRFI1 knockdown reduced apoptotic and ferroptotic hepatocytes induced by OGD/R. Mechanistically, ERRFI1 interacted with GRB2 to maintain its stability by hindering its proteasomal degradation. Overexpression of GRB2 abrogated the effects of ERRFI1 silencing on hepatocyte apoptosis and ferroptosis.

**Conclusions:**

Our results revealed that the ERRFI1-GRB2 interaction and GRB2 stability are essential for ERRFI1-regulated hepatic IR injury, indicating that inhibition of ERRFI1 or blockade of the ERRFI1-GRB2 interaction may be potential therapeutic strategies in response to hepatic IR injury.

**Supplementary Information:**

The online version contains supplementary material available at 10.1186/s10020-024-00837-4.

## Introduction

Hepatic ischemia and reperfusion (IR) injury results from blood deprivation followed by reperfusion, and occurs in a variety of clinical situations including hepatic resection surgery, liver transplantation, hemorrhagic shock, and other systemic low-flow diseases (Saidi and Kenari 2014; Mao et al. 2021). Local hepatocellular damage during IR involves interactions between hepatocytes, hepatic sinusoidal endothelial cells, Kupffer cells, and hepatic stellate cells, as well as infiltrating neutrophils, macrophages and platelets (Peralta et al. 2013). IR poses injuries to hepatic tissue through reduced ATP availability, a burst of ROS generation, enhanced inflammatory response, and initiation of cell death (Kalogeris et al. 2014). Apoptosis is a typical dead form of cell loss and accounts for a large proportion of cell death following hepatic IR (Jaeschke and Lemasters 2003). Ferroptosis, a novel type of cell death in hepatic IR injury, is driven by iron and lipid peroxidation-dependent regulation (Dixon et al. 2012). Therefore, a thorough understanding of IR injury mechanisms (such as apoptosis and ferroptosis) is of great importance to develop new treatment strategies and ameliorate liver injury in hepatic IR patients.

ERBB receptor feedback inhibitor 1 (ERRFI1, also known as MIG6, RALT, GENE-33) encodes a 50 kDa cytoplasmic transition protein that is rapidly induced by a wide range of mitogenic, endocrine and stress signals (Makkinje et al. 2000; Xu et al. 2005). It suppresses the catalytic activity of EGFR by disrupting its dimerization and acts as an important player in the attenuation of the EGFR signaling network (Izumchenko and Sidransky 2015). ERRFI1 localizes in human chromosome 1p36, a locus that has been widely suggested to harbor putative tumor-suppressor genes (Zhang et al. 2007). Previous studies have demonstrated that ERRFI1 serves as a tumor suppressor gene with reduced expression in a variety of human cancers, and loss of function of this protein contributes to cancer development (Zhang et al. 2007; Amatschek et al. 2004; Cui et al. 2022). For example, ERRFI1 induces apoptosis of hepatocellular carcinoma cells (Cui et al. 2021; Qu et al. 2022). In addition to cancer, ERRFI1 has been implicated in many pathological situations. Bellini et al. indicated that overexpression of ERRFI1 in cartilage triggers an osteoarthritis-like phenotype in mice (Bellini et al. 2020). It has been demonstrated that ERRFI1 is induced by hypoxia in cardiomyocytes and ischemia/reperfusion in myocardial tissues, and promotes apoptosis of cardiomyocytes (Xu et al. 2006). Regarding the expression and function of ERRFI1 in the normal liver, its expression level in the liver is high, and it plays an important role in cholesterol homeostasis and bile acid synthesis under normal physiological conditions (Reschke et al. 2010; Ku et al. 2012). Nevertheless, the role of ERRFI1 under hepatic IR conditions has not been investigated.

Studies have confirmed that ferroptosis is involved in hepatic IR injury (Yamada et al. 2020; Guo et al. 2022; Wu et al. 2022). Yamada et al. demonstrated that ferroptosis is induced by iron overload in donor serum obtained from hepatic IR injury after liver transplantation, and treatment with a ferroptosis-specific inhibitor in a mouse model of hepatic IR inhibited liver damage and lipid peroxidation (Yamada et al. 2020). By inducing ubiquitination and degradation of GPX4, GPX4-dependent ferroptosis is enhanced in hepatic IR injury (Guo et al. 2022). These results imply that ferroptosis may be a therapeutic target for hepatic IR injury, although the mechanisms regulating ferroptosis in hepatic IR injury are not completely understood.

To date, there are no reports on whether ERRFI1 is involved in hepatic IR injury by regulating apoptosis and ferroptosis of hepatocytes. In this study, we aimed to investigate the regulatory mechanism of ERRFI1 on liver injury through the modulation of apoptosis and ferroptosis.

## Material and method

### Animals

Hepatocyte-specific ERRFI1 knockout (ERRFI1-HKO) mice were generated by mating ERRFI1-flox mice (Cyagen Biosciences) with Albumin (Alb)-enhancer/promoter-driven Cre transgenic mice (GemPharmatech Co., Ltd.). C57BL/6J wild-type (WT) mice (Liaoning Changsheng biotechnology Co., Ltd.) and ERRFI1-HKO mice were housed in a specific pathogen-free facility (temperature of 24 ± 2 °C, humidity between 30 and 70%) with alternating 12:12 h light-dark cycles and were allowed access to food and water ad libitum.

### Mouse model of hepatic IR

All animal experiments were approved by the Animal Care and Use Committee of The First Affiliated Hospital of Jinzhou Medical University. The experiments were conducted in accordance with the National Institutes of Health (NIH) Guide for the Care and Use of Laboratory Animals. Following anesthesia, 8- to 10-week-old mice were incised at the ventral midline to expose the liver hilum. A noninvasive vascular clamp was employed to interrupt the hepatic artery/portal venous blood supply to the left and middle lobes. After 90 min of ischemia, the clamp was removed to restore hepatic blood flow, and the abdomen was closed with sutures. To maintain body temperature throughout the surgical procedures, mice were placed supine on a heating pad under a heat lamp. No hepatic blood flow blockage was performed in Sham mice. Blood samples and ischemic hepatic tissues were collected at 6 h of reperfusion.

### Biochemical analysis

Blood samples were centrifuged to obtain serum. The degree of hepatic injury was detected by the serum levels of alanine aminotransferase (ALT) and aspartate aminotransferase (AST) using the ALT Activity Assay Kit (ab105134; Abcam, Cambridge, UK) and the AST Activity Assay Kit (ab138878; Abcam).

The levels of glutathione (GSH) and malondialdehyde (MDA) in liver tissues and hepatocytes were measured using commercial kits (A006, A003; Jiancheng Bioengineering Institute, Nanjing, China) according to the manufacturer’s instructions.

### Hematoxylin and eosin (H&E) staining

Liver tissues were fixed with paraformaldehyde (Sinopharm Chemical Reagent Beijing Co., Ltd., Beijing, China), and dehydrated with gradient alcohol. Afterwards, the tissues were embedded in paraffin (M060590, MREDA) and sectioned into 5-µm slices. The slices were deparaffinized with xylene (Sinopharm Chemical Reagent Beijing Co., Ltd.) and then rehydrated in gradient alcohol. The sections were stained with hematoxylin (Sigma, St Louis, MO, USA), incubated in hydrochloric acid solution (Sinopharm Chemical Reagent Beijing Co., Ltd.), and stained with eosin (Xiya Reagent, Linyi, China). After dehydration and sealing with neutral gum (Solarbio Science & Technology, Co., Ltd., Beijing, China), the sections were observed under a microscope (OLYMPUS, Tokyo, Japan) at a magnification of 200×. The histological severity of hepatic injury was graded using Suzuki’s method. The Suzuki criteria are as follows: congestion (none = 0, minimal = 1, mild = 2, moderate = 3, severe = 4), vacuolization (none = 0, minimal = 1, mild = 2, moderate = 3, severe = 4) and necrosis (none = 0, single cell necrosis = 1, < 30% = 2, 30–60% = 3, > 60% = 4); scores for each parameter ranged from 0 to 4, with a maximum score of 12.

### Terminal deoxynucleotidyl transferase-mediated dUTP nick end labeling (TUNEL) staining

For liver tissues, the sections were dewaxed and rehydrated. For cell slides, the slides were fixed with paraformaldehyde. A TUNEL Cell Apoptosis Detection Kit (G1504; Servicebio, Wuhan, China) was employed to determine the apoptotic cells. The images were photographed using a microscope (OLYMPUS) at a magnification of 400×.

### Western blot analysis

Total proteins from liver tissues and hepatocytes were extracted by RIPA lysis buffer (AS1004; Aspen Biological, Wuhan, China). The protein concentration in the supernatants was measured using a BCA protein assay kit (AS1086, Aspen Biological). Proteins were loaded on sodium dodecyl sulfate-polyacrylamide gels and transferred to PVDF membranes. After blocking, membranes were incubated with primary antibodies overnight at 4℃, followed by horseradish peroxidase-conjugated secondary antibodies (Aspen Biological). Finally, protein bands were visualized by an enhanced chemiluminescence kit (AS1059, Aspen Biological). The following primary antibodies were used: antibodies for ERRFI1 (sc-137,154; Santa Cruz Biotechnology, Santa Cruz, CA, USA), Bax (#2772; Cell Signaling Technology, Danvers, MA, USA), Bcl-2 (ab182858, Abcam), cleaved caspase-3 (AF7022; Affinity Biosciences, Cincinnati, OH, USA), caspase-3 (AF6311, Affinity Biosciences), GRB2 (ab32037, Abcam), and GAPDH (ab181602, Abcam).

### ROS measurement

ROS production was evaluated using the ROS sensitive fluorescent probe DCFH-DA (S0033; Beyotime Institute of Biotechnology, Haimen, China). Liver homogenate or hepatocytes were incubated with DCFH-DA probe at 37℃ for 20 min. After washing with serum-free solution three times, cell samples were analyzed using a fluorescence microplate reader.

### Ferrous iron (Fe^2+^) measurement

An Iron Assay Kit (ab83366, Abcam) was employed to measure the iron concentration in liver homogenate or cell lysates. Briefly, assay buffer was added to liver homogenate or cell lysates and incubated for 30 min at 37℃, then iron probe was added and incubated for 60 min at 37℃. The absorbance at 593 nm was measured using a microplate reader. An iron standard curve was constructed using known concentrations of iron solutions. The iron concentration was normalized to the protein concentration.

### Real-time PCR analysis

RNAiso Plus (9109; Takara, Tokyo, Japan) was employed to isolate total RNA from liver tissues or hepatocytes, and the PrimeScript™ RT reagent Kit (RR047A, Takara) was used to synthesize cDNA. Quantitative real-time PCR was carried out with SYBR Green PCR Master Mix (QPK-201; TOYOBO, Osaka, Japan). The relative gene-expression was normalized to the RNA level of the housekeeping gene β-actin, and the data were calculated according to the 2^−ΔΔCt^ method. The primer sequences were as follows: mouse ACSL4: Forward 5’-CTTCCTCTTAAGGCCGGGAC-3’, Reverse 5’-TGCCATAGCGTTTTTCTTAGATTT-3’; mouse SLC7A11: Forward 5’-TTGCAAGCTCACAGCAATTC-3’, Reverse 5’-AGGGCAACCCCATTAGACTT-3’; mouse GPX4: Forward 5’-TGCATCGTCACCAACGTGGC-3’, Reverse 5’-CTTCACCACGCAGCCGTTCT-3’; human ACSL4: Forward 5’-GGAATGACAGGCCAGTGTGA-3’, Reverse 5’-TAGCACATGAGCCAAAGGCA-3’; human SLC7A11: Forward 5’-GGGCATGTCTCTGACCATCT-3’, Reverse 5’-TCCCAATTCAGCATAAGACAAA-3’; human GPX4: Forward 5’-CGATACGCTGAGTGTGGTTTGC-3’, Reverse 5’-CATTTCCCAGGATGCCCTTG-3’; human ERRFI1: Forward 5’-CTGGAGCAGTCGCAGTGAG-3’, Reverse 5’-GCCATTCATCGGAGCAGATTTG-3’; and human GRB2: Forward 5’-CCATCGCCAAATATGACTTCAAA-3’, Reverse 5’-CTTCGTTCAAAACCTTGAGGATGT-3’.

### Immunohistochemical staining

Liver sections were incubated with 3% H_2_O_2_ (Sinopharm Chemical Reagent Beijing Co., Ltd.) to block endogenous peroxidase. Then the sections were incubated with primary antibodies diluted in bovine serum albumin (BSA; Biofroxx, Einhausen, Germany) overnight at 4℃. Primary antibodies were: 8-OHdG (ab48508; Abcam), ACSL4 antibody (22401-1-AP; Proteintech Group, Wuhan, China), SLC7A11 antibody (26864-1-AP, Proteintech Group), and GPX4 antibody (67763-1-Ig, Proteintech Group). After washing with PBS, the sections were incubated with the goat anti-rabbit/mouse IgG secondary antibodies (Aspen Biological) at 37℃ for 30 min. Subsequently, the sections were stained with 3,3’-diaminobenzidine (DAB; ZLI-9019; ZSGB-Bio, Beijing, China) and counterstained with hematoxylin (Sigma). The sections were subjected to dehydration and sealing, and were observed under a microscope (OLYMPUS) at a magnification of 400×.

### Cell culture and treatments

The normal human liver cell line L-02 was purchased from iCell Bioscience Inc (Shanghai, China). L-02 cells were cultured in RPMI-1640 medium (iCell Bioscience Inc) containing 20% fetal bovine serum (FBS) and 1% penicillin-streptomycin mixture and maintained in a humidified incubator with 5% CO_2_ at 37℃.

For cell treatments, L-02 cells were treated with 10 µM MG132 (S1748, Beyotime Institute of Biotechnology) for 4 h, or treated with 100 µg/mL cycloheximide (CHX; ab120093, Abcam) for 0, 3, 6, or 9 h.

### Cell transfection

Specific shRNA oligonucleotides targeting ERRFI1 mRNA and nontargeting version of shRNA were inserted into the pRNA-H1.1 plasmid to construct sh-ERRFI1 and sh-NC vectors. The PCR-amplified coding sequence of GRB2 was cloned into the pCDNA3.1(+) plasmid to generate GRB2 overexpression plasmid. L-02 cells were transfected with the above vectors using Lipofectamine 3000 (Invitrogen, Carlsbad, CA, USA) according to the manufacturer’s instructions.

### Oxygen-glucose deprivation and reoxygenation (OGD/R) model

For oxygen-glucose deprivation experiments, normal culture medium was replaced with glucose-free RPMI-1640 medium. Cells in this group were incubated in a hypoxic environment with 5% CO_2_, 1% O_2_, and 94% N_2_ in a 37℃ incubator for 4 h. For reoxygenation, the glucose-free medium was then replaced with normal culture medium, and the cells were cultured under normal conditions (95% air, 5% CO_2_) for 12 h.

### CCK-8 assay

L-02 cells were seeded in 96-well plates at a density of 5 × 10^3^ cells/well. After OGD/R treatment, 10 µL CCK8 reagent (C0042, Beyotime Institute of Biotechnology) was added to each well and incubated at 37℃ for 4 h. The absorbance was detected at 450 nm using a microplate reader (Bio-Rad; Hercules, CA, USA).

### Determination of lipid peroxidation

To determine OGD/R-induced lipid peroxidation in vitro, L-02 cells with different treatment were collected. After washing with PBS, 10 µM C11-BODIPY 581/591 (Invitrogen; Carlsbad, CA, USA) was added to cell medium and incubated at 37℃ for 1 h. The nuclei were labeled with Hoechst 33,258 (Solarbio Science & Technology, Co., Ltd.). Fluorescence intensity was measured using flow cytometry (BD; Franklin Lakes, NJ, USA).

### Immunofluorescence staining

To perform immunofluorescence staining, cell slides were prepared. The slides were fixed with 4% paraformaldehyde. After being washed with PBS, the slides were incubated with primary antibody against GPX4 (67763-1-Ig), ACSL4 antibody (22401-1-AP), and SLC7A11 antibody (26864-1-AP) overnight at 4℃. The slides were then washed, and incubated with the secondary antibody at 37℃ for 40 min. Nuclear staining was conducted using 4’,6-diamidino-2-phenylindole (DAPI, Sigma) at room temperature for 30 min in the dark. The slides were observed under a fluorescence microscope (OLYMPUS) at a magnification of 400×.

### Propidium iodide (PI) staining

Propidium iodide (PI; P8080, Solarbio Science & Technology, Co., Ltd.) was dissolved in DMSO at a final concentration of 1.5 mM. L-02 cells with different treatment were fixed with 4% paraformaldehyde and stained with PI dye in the dark for 10 min. After removal of PI dye and being washed with PBS, the cells were covered with antifade mounting medium and observed under a fluorescence microscope (OLYMPUS) at a magnification of 200×.

### Co-immunoprecipitation (Co-IP)

Cells were lysed with lysis buffer on ice. Then cell lysates were adjusted to equal amounts of protein and immunoprecipitated with GRB2 antibody (ab32037, Abcam) at 4℃ overnight. Rabbit IgG control antibody (#3423; Cell Signaling Technology, Danvers, MA, USA) was used as a negative control. The immunocomplex was pulled down with Protein A/G PLUS-Agarose (sc-2003, Santa Cruz Biotechnology). Immunoprecipitates were subjected to SDS-PAGE, and coimmunoprecipitated proteins were detected by immunoblotting with GRB2 antibody (ab32037, Abcam) or ERRFI1 (sc-137,154, Santa Cruz Biotechnology).

### Statistical analysis

Data are expressed as the mean ± standard deviation (SD). Analysis was performed using GraphPad Prism 9.0. Data were analyzed using Student’s t test and one-way analysis of variance (ANOVA) followed by Tukey’s multiple comparisons test, and a *p* value < 0.05 was considered statistically significant.

## Results

### ERRFI1 deficiency ameliorated IR-induced hepatic injury and hepatocellular apoptosis

**Fig. 1:**
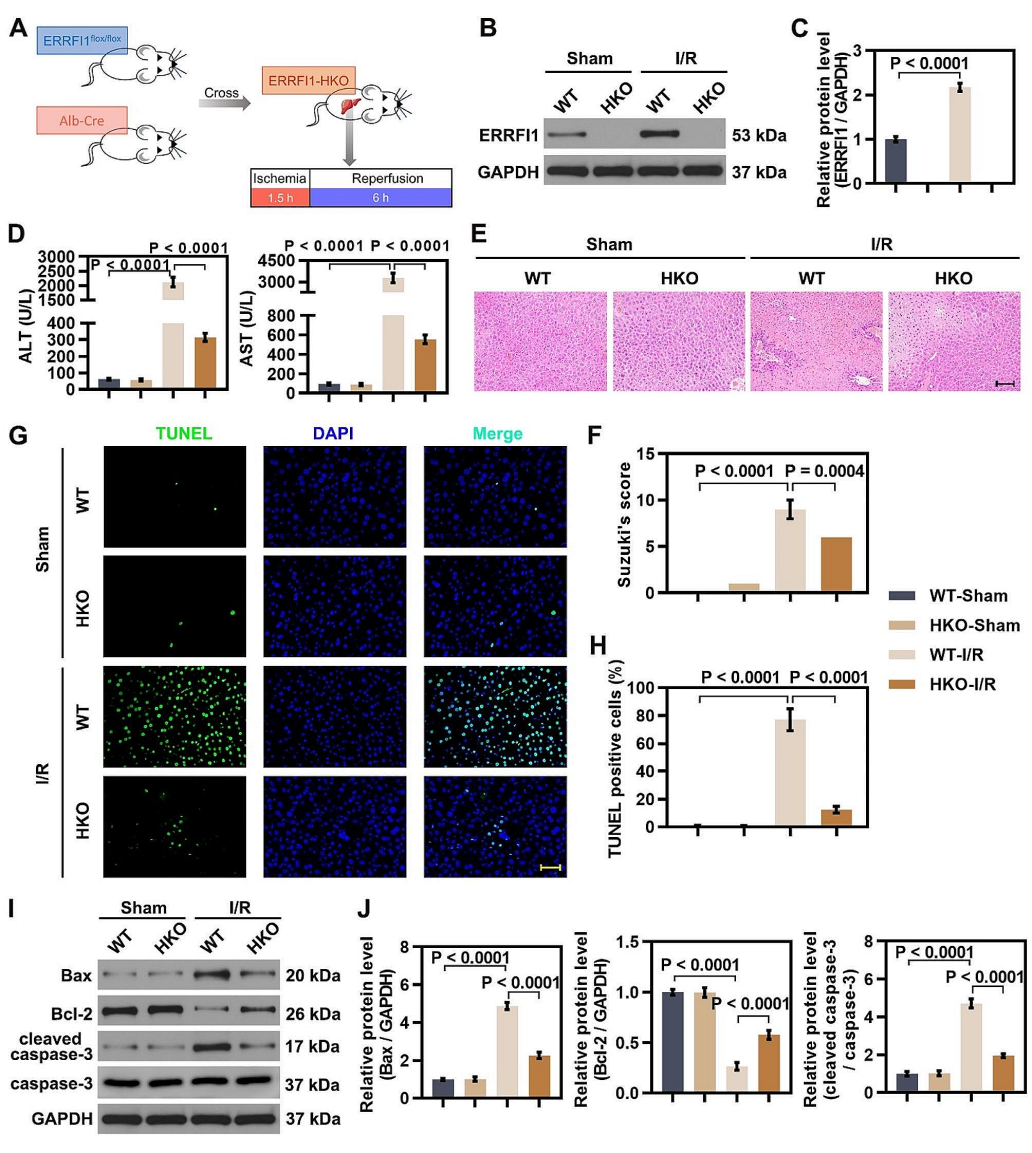
ERRFI1 deficiency ameliorated IR-induced hepatic injury and hepatocellular apoptosis. (**A**) Schematic diagram showed the hepatocyte-specific ERRFI1 knockout strategy and ischemia reperfusion model. (**B, C**) Western blot analysis of ERRFI1 protein level in liver tissues from wild type (WT) mice and hepatocyte-specific ERRFI1-knockout (ERRFI1-HKO) mice with sham treatment or ischemia for 90 min followed by reperfusion for 6 h, and quantitative analysis is shown. (**D**) Liver function assessed by ALT and AST of mice with different treatment. (**E**) Liver pathology was determined by H&E staining (scale bar: 100 μm). (**F**) Suzike’s injury score was used to assess the degree of injury based on H&E staining. (**G, H**) TUNEL staining of apoptotic cells in liver tissues from WT mice and ERRFI1-HKO mice under different conditions (scale bar: 50 μm), and quantification showing the percentage of apoptotic cells. (**I, J**) Western blot analysis of Bax, Bcl-2, and cleaved caspase-3 in liver tissues from WT mice and ERRFI1-HKO mice after IR injury. For statistical analysis, one-way ANOVA was used (*n* = 6)

To explore the potential involvement of ERRFI1 in hepatic IR injury, hepatocyte-specific ERRFI1-knockout (herein, ERRFI1-HKO) mice and wild type (herein, WT) control mice were subjected to IR challenge (Fig. 1A). The ERRFI1 knockout efficiency was verified western blot analysis (Fig. 1B, C). First, serum ALT and AST levels were measured to determine the effect of ERRFI1 deficiency on hepatic IR injury. Under sham-operated conditions, serum AST and ALT levels were comparable in ERRFI1-HKO mice and WT mice, suggesting that ERRFI1 deficiency had no effect on hepatic damage. Notably, the levels of ALT and AST in ERRFI1-HKO mice were reduced compared with those in WT mice after hepatic IR injury (Fig. 1D). In H&E staining of liver sections, IR-treated WT mice exhibited severe structural liver damage with wide necrosis of hepatocytes and infiltration of inflammatory cells, while ERRFI1-HKO mice displayed relatively well-preserved histological architecture with less hepatocyte damage upon hepatic IR (Fig. 1E, F). Next, we analyzed hepatocellular apoptosis in ischemic livers by TUNEL assay. ERRFI1-HKO mice showed fewer TUNEL-positive cells than WT mice after exposure to hepatic IR injury (Fig. 1G, H). Western blot analysis revealed that the expression of Bax and cleaved caspase-3 was reduced and the expression of Bcl-2 was increased in IR-stressed livers from ERRFI1-HKO mice compared with WT mice (Fig. 1I, J). Taken together, these results indicated that ERRFI1 deficiency relieved IR-induced hepatic injury and hepatocellular apoptosis.

### ERRFI1 deficiency protected against hepatic IR-induced ferroptosis

**Fig. 2:**
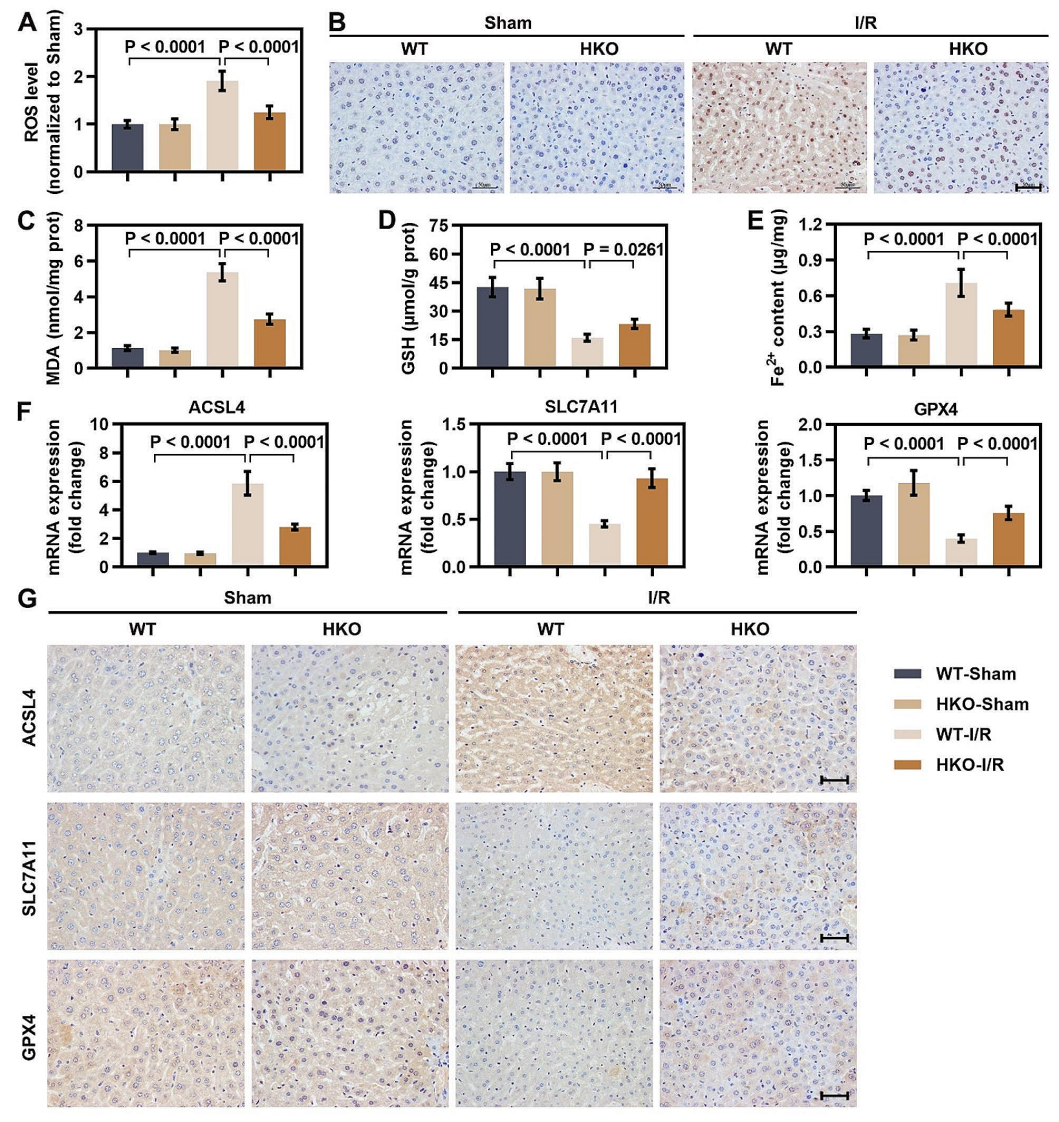
ERRFI1 deficiency protected against hepatic IR-induced ferroptosis. (**A**) Intracellular ROS level was determined by DCFH-DA staining after hepatic IR. (**B**) Immunohistochemical staining of 8-OHdG in liver tissues (scale bar: 50 μm). (**C, D**) The content of MDA and the level of GSH in the livers of mice subjected to sham treatment or to an induction of IR. (**E**) Hepatic Fe^2+^ content in each group. (**F**) The mRNA levels of ACSL4, SLC7A11, and GPX4 in liver tissues of mice with different treatments. (**G**) Representative immunohistochemical images of ACSL4, SLC7A11, and GPX4 in liver tissues (scale bar: 50 μm). For statistical analysis, one-way ANOVA was used (*n* = 6)

To examine the regulation of ferroptosis by ERRFI1 in the process of hepatic IR, we detected ROS levels in liver tissues by using DCFH-DA probe. Although livers from WT and ERRFI1-HKO mice had similar ROS levels after the sham procedure, livers from WT mice had much higher ROS generation after IR, and ERRFI1 deficiency inhibited IR-induced excessive ROS production (Fig. 2A). Excessive ROS accumulation results in DNA damage, and 8-OHdG is a marker of oxidative DNA damage. Therefore, the expression of 8-OHdG in liver tissues was investigated. The results indicated that the level of 8-OHdG in WT mice was increased after IR, and 8-OHdG expression was decreased by ERRFI1 deficiency under IR conditions (Fig. 2B). Next, the content of MDA (a secondary product of lipid peroxidation) in liver tissues was determined. By 6 h of reperfusion, the level of MDA was lower in ERRFI1-HKO mice than in WT mice (Fig. 2C). IR repressed the antioxidant GSH in liver tissue, while the hepatic level of GSH was higher in ERRFI1-HKO mice than in WT mice under IR conditions (Fig. 2D). Massive Fe^2+^ content in the liver tissues of WT mice was observed after 6 h of reperfusion, suggesting that ferroptosis occurred after IR. However, a minor Fe^2+^ content was detected in the livers of ERRFI1-HKO mice subjected to IR (Fig. 2E). In liver tissues from WT mice, the mRNA levels of negative regulators of ferroptosis (SLC7A11 and GPX4) were reduced after IR, while the mRNA level of the positive regulator of ferroptosis (ACSL4) was increased. However, these expression changes were reversed in ERRFI1-HKO mice (Fig. 2F). Immunohistochemical staining confirmed that the changes in the protein expression of the above ferroptosis markers were consistent with the changes in mRNA expression (Fig. 2G). These results suggested that ERRFI1 deficiency alleviated ferroptosis in hepatic IR injury.

### Knockdown of ERRFI1 inhibited apoptosis of hepatocytes induced by hypoxic-reoxygenation

**Fig. 3:**
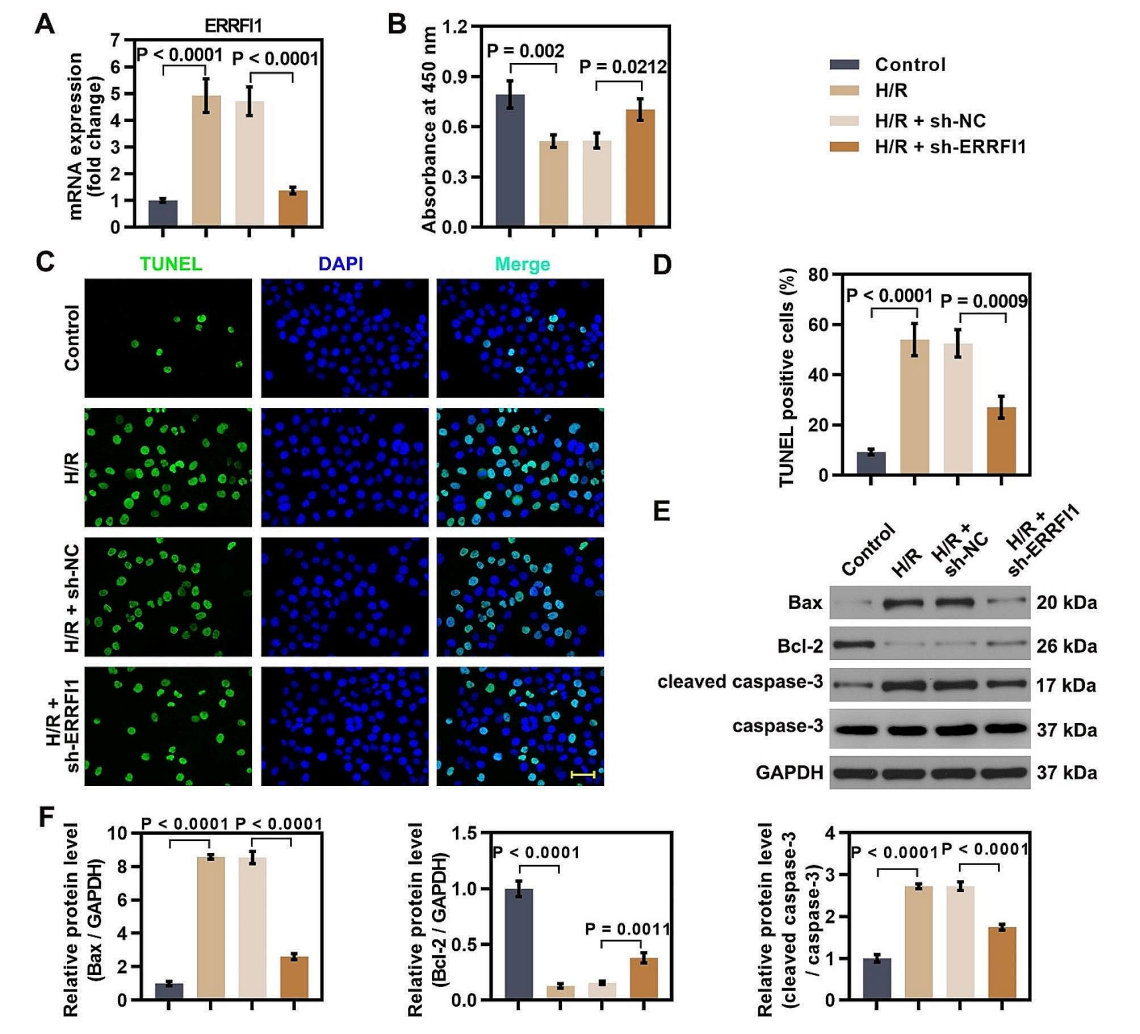
Knockdown of ERRFI1 inhibited apoptosis of hepatocytes induced by hypoxic-reoxygenation. (**A**) Expression of ERRFI1 in L-02 cells following hypoxia/reoxygenation (H/R) and ERRFI1 knockdown at the mRNA level was determined by real-time PCR. (**B**) Cell viability of ERRFI1-silenced L-02 cells after OGD/R exposure. (**C, D**) Apoptosis-positive cells were detected by TUNEL staining (scale bar: 50 μm). (**E, F**) Western blot analysis of Bax, Bcl-2, and cleaved caspase-3 in L-02 cells under indicated conditions. For statistical analysis, one-way ANOVA was used (*n* = 3)

To explore the cellular function of ERRFI1 in hepatic damage, we constructed an oxygen-glucose deprivation/reoxygenation (OGD/R) model in hepatocytes in vitro to mimic the hepatic IR environment in vivo. OGD/R stimulation led to increased expression of ERRFI1 in hepatocytes, and knockdown of ERRFI1 by transfection with ERRFI1-shRNA was successful under OGD/R conditions (Fig. 3A). ERRFI1 knockdown restored the decreased cell viability in OGD/R-induced hepatocytes (Fig. 3B). The OGD/R-induced increase in TUNEL-positive cells was decreased by ERRFI1 knockdown (Fig. 3C, D). The expression of Bax and cleaved caspase-3 was increased, and the expression of Bcl-2 was reduced in OGD/R-induced hepatocytes. However, ERRFI1 silencing reversed the expression changes in apoptosis-related markers (Fig. 3E, F). The results indicated that knockdown of ERRFI1 attenuated apoptosis in OGD/R-induced hepatocytes.

### Knockdown of ERRFI1 suppressed OGD/R-induced ferroptosis in hepatocytes

**Fig. 4:**
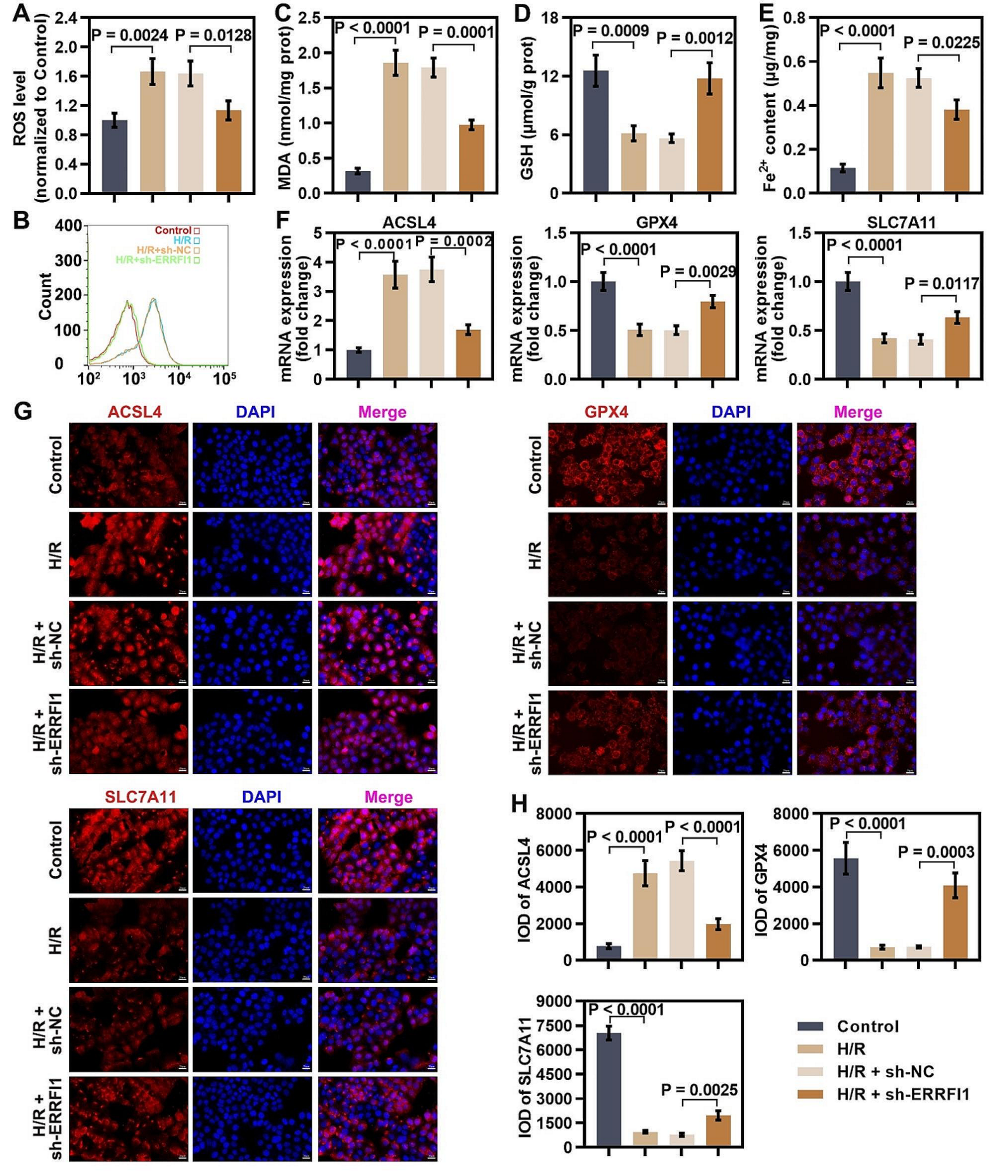
Knockdown of ERRFI1 suppressed OGD/R-induced ferroptosis in hepatocytes. (**A**) ROS level in ERRFI1-silenced L-02 cells exposed to hypoxia/reoxygenation. (**B**) Flow cytometry analysis of lipid peroxidation using C11-BODIPY 581/591 in L-02 cells under indicated conditions. (**C, D**) MDA content and GSH level in L-02 cells cultured under indicated conditions. (**E**) Fe^2+^ content in ERRFI1-silenced L-02 cells after OGD/R exposure was determined. (**F**) Real-time PCR showed the mRNA levels of ACSL4, SLC7A11, and GPX4 in response to ERRFI1 knockdown under H/R conditions. (**G, H**) Fluorescence immunostaining of ACSL4, SLC7A11, and GPX4 in L-02 cells transfected with sh-ERRFI1 during H/R injury (scale bar: 20 μm). For statistical analysis, one-way ANOVA was used (*n* = 3)

Based on the in vivo results, we further validated whether ERRFI1 deficiency could prevent OGD/R-induced ferroptosis in hepatocytes. The DCFH-DA fluorescent probe was used to evaluate intracellular ROS levels in hepatocytes after OGD/R. The results demonstrated that knockdown of ERRFI1 diminished ROS production induced by OGD/R stimulation (Fig. 4A). To further determine lipid peroxidation, lipid ROS level was measured using C11–BODIPY staining. We found that OGD/R stimulation increased lipid ROS production in hepatocytes. However, ERRFI1 knockdown reduced lipid ROS production induced by OGD/R (Fig. 4B). ERRFI1 deficiency inhibited OGD/R-induced increase in the content of MDA and Fe^2+^, and prevented the reduction in GSH level (Fig. 4C-E). OGD/R treatment gave rise to the upregulation of ACSL4 and downregulation of SLC7A11 and GPX4, whereas ERRFI1-shRNA transfection reversed the effect of OGD/R induction on the changes in mRNA and protein expression of these ferroptosis markers (Fig. 4F-H). All these data revealed that knockdown of ERRFI1 suppressed OGD/R-induced ferroptosis in hepatocytes.

### ERRFI1 directly interacted with GRB2 and maintained its stability by hindering its proteasomal degradation

**Fig. 5:**
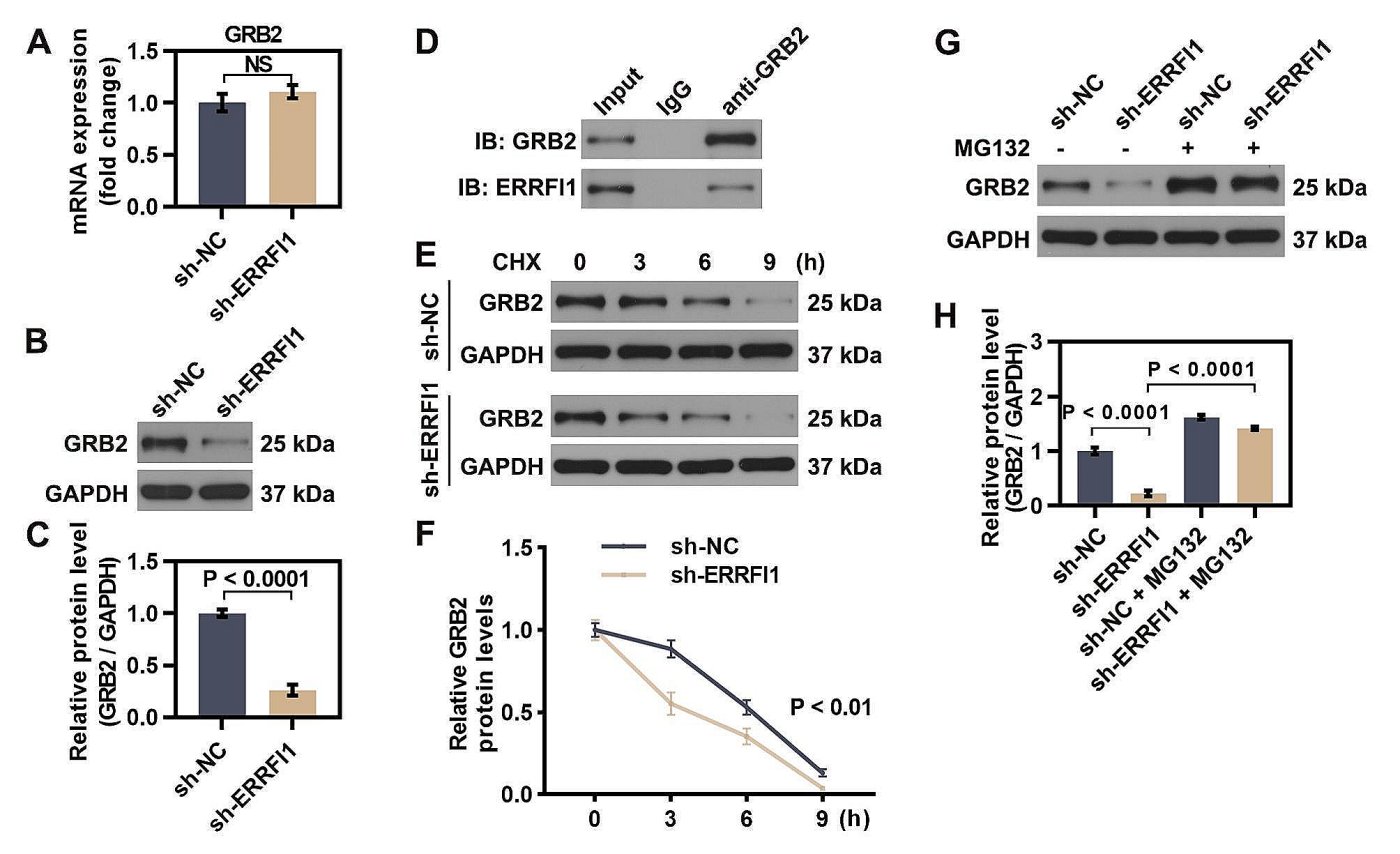
ERRFI1 directly interacted with GRB2 and maintained its stability by hindering its proteasomal degradation. (**A-C**) Transcript and protein levels of GRB2 in ERRFI1-silenced L-02 cells were detected. (**D**) Co-immunoprecipitation of ERRFI1 and GRB2. L-02 cells were subjected to GRB2 immunoprecipitation and subsequent immunoblotting of ERRFI1 and GRB2. (**E, F**) L-02 cells were transfected with sh-ERRFI1 and treated with CHX for the indicated times. Western blot analysis showed the expression of GRB2. (**G, H**) The expression of GRB2 in sh-ERRFI1-transfected L-02 cells with or without MG132 treatment. For statistical analysis, Student’s t test was used (*n* = 3)

ERRFI1 is able to bind to proteins, and has been shown to interact with GRB2 (growth factor receptor-bound protein 2) according to the Biogrid website (https://thebiogrid.org/). Furthermore, based on the evidence that GRB2 contributes to hepatocyte apoptosis in non-alcoholic fatty liver disease (Shan et al. 2013), we proposed that GRB2 may act as a direct target of ERRFI1 to mediate apoptosis and ferroptosis in hepatic IR injury. Therefore, we began by exploring the relationship between ERRFI1 and GRB2 in hepatocytes. Intriguingly, knockdown of ERRFI1 did not alter the mRNA level of GRB2 in hepatocytes, but decreased the protein expression of GRB2 (Fig. 5A-C), suggesting the post-translational regulation of GRB2 expression by ERRFI1. Co-IP experiment further demonstrated that ERRFI1 interacted with GRB2 in hepatocytes (Fig. 5D). Following CHX (an inhibitor of protein synthesis) treatment, knockdown of ERRFI1 led to much faster degradation of GRB2 in hepatocytes (Fig. 5E, F), indicating that ERRFI1 maintained the protein stability of GRB2. Moreover, GRB2 degradation driven by ERRFI1-shRNA was rescued by the proteasome-specific inhibitor MG132 in hepatocytes, suggesting that ERRFI1 suppressed GRB2 degradation by disturbing the proteasomal pathway (Fig. 5G, H). These results implied that ERRFI1 maintained the stability of GRB2 in hepatocytes by hindering its proteasomal degradation.

### ERRFI1 facilitated OGD/R-induced injury of hepatocytes in a GRB2-dependent manner

**Fig. 6:**
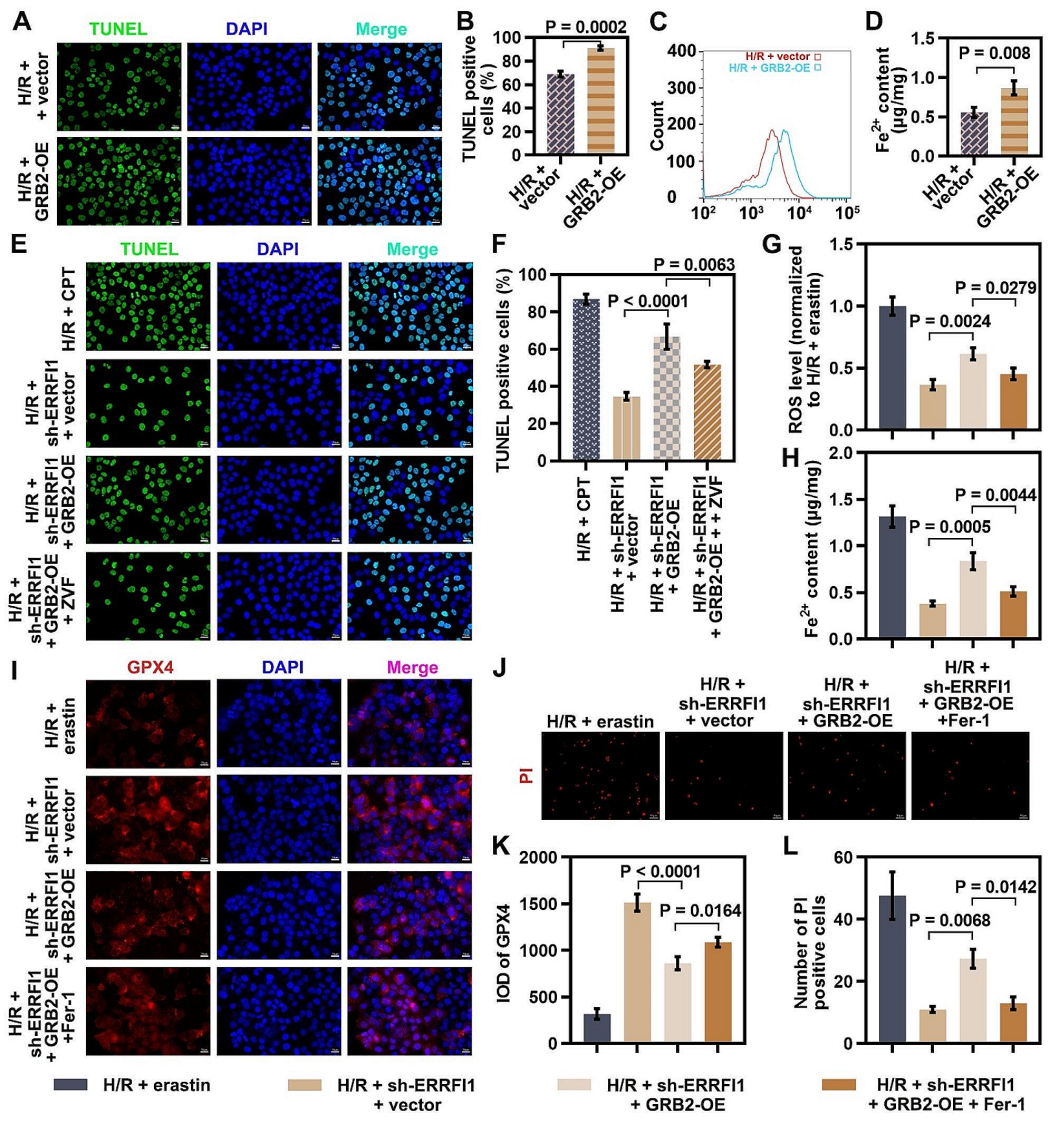
ERRFI1 facilitated OGD/R-induced injury of hepatocytes in a GRB2-dependent manner. (**A-D**) L-02 cells were transfected with GRB2 overexpression plasmid or vector plasmid, followed by 4 h of hypoxia and 12 h of reoxygenation. Cell apoptosis was detected by TUNEL staining. Scale bar: 20 μm (**A, B**). Lipid peroxidation was measured using C11-BODIPY 581/591 (**C**). Fe^2+^ content was determined (**D**). (**E, F**) L-02 cells cotransfected with sh-ERRFI1 and GRB2 overexpression plasmid were subjected to OGD/R stimulation in the presence or absence of 20 µM Z-VAD-FMK (ZVF, an apoptosis inhibitor). L-02 cells were treated with 5 µM camptothecin (CPT, an apoptosis inducer) as positive control at the same time as OGD/R stimulation. TUNEL staining of L-02 cells under indicated conditions was performed (scale bar: 20 μm). (G-L) L-02 cells cotransfected with sh-ERRFI1 and GRB2 overexpression plasmid were subjected to OGD/R stimulation in the presence or absence of 5 µM ferrostatin-1 (Fer-1, a ferroptotic inhibitor). L-02 cells were treated with 10 µM erastin (a ferroptotic inducer) as positive control at the same time as OGD/R stimulation. DCFH-DA staining was used to detect ROS production in cells (**G**). Fe^2+^ content in cells was measured by a commercial kit (**H**). The protein expression of GPX4 in cells was analyzed by immunofluorescence. Scale bar: 20 μm (**I, K**). Cell death was detected by propidium iodide (PI) staining. Scale bar: 50 μm (J, L). For statistical analysis, student’s t test and one-way ANOVA were used (*n* = 3)

Subsequently, we investigated the role of GRB2 in OGD/R-induced injury of hepatocytes. It was observed that GRB2 overexpression promoted apoptosis, enhanced lipid ROS level, and increased Fe^2+^ content in hepatocytes under OGD/R stimulation (Fig. 6A-D), suggesting GRB2 facilitated apoptosis and ferroptosis of OGD/R-induced hepatocytes.

To further clarify whether ERRFI1 mediates OGD/R-induced apoptosis of hepatocytes via the regulation of GRB2, ERRFI1-shRNA and GRB2 overexpression plasmid were cotransfected into OGD/R-induced hepatocytes in the presence or absence of Z-VAD-FMK (an apoptosis inhibitor). Camptothecin (CPT), an inhibitor of DNA topoisomerase-I, was used as a positive control for apoptosis induction. Upon OGD/R induction, GRB2 overexpression promoted cell apoptosis in ERRFI1-deficient hepatocytes. However, Z-VAD-FMK treatment abrogated GRB2 overexpression-triggered apoptosis in ERRFI1-deficient hepatocytes (Fig. 6E, F). To further verify the effect of the ERRFI1/GRB2 axis on ferroptosis in OGD/R-induced hepatocytes, hepatocytes cotransfected with ERRFI1-shRNA and GRB2 overexpression plasmid were subjected to OGD/R stimulation and treated with a ferroptotic inhibitor (ferrostatin-1). Erastin, a synthetic compound that inhibits cystine/glutamate antiporters and causes ferroptosis, was used as a positive control for ferroptosis induction. Under OGD/R conditions, GRB2 overexpression abrogated the inhibition of these ferroptotic events in ERRFI1-deficient hepatocytes, as evidenced by enhanced ROS level, elevated Fe^2+^ content, reduced GPX4 expression, and increased cell death. However, ferrostatin-1 treatment reversed ERRFI1/GRB2 axis-triggered ferroptosis in hepatocytes (Fig. 6G-L). These findings suggested that ERRFI1 facilitated OGD/R-induced apoptosis and ferroptosis of hepatocytes in a GRB2-dependent manner.

## Discussion

The present study provided evidence that ERRFI1 expression was increased in liver tissues from mice with IR injury. In a mouse model, hepatocyte-specific knockout of ERRFI1 attenuated excessive apoptosis and ferroptosis upon IR injury. The effects of ERRFI1 deficiency in human hepatocytes under OGD/R conditions was in agreement with the results obtained in vivo. Further investigation demonstrated that ERRFI1 directly interacted with GRB2 and mediated its proteasomal degradation. Importantly, overexpression of GRB2 reversed the potentiated effects of ERRFI1 deficiency in OGD/R-induced apoptosis and ferroptosis, indicating that targeting ERRFI1 or blocking the ERRFI1-GRB2 axis may represent promising approaches to alleviate hepatic IR injury.

A large and growing body of literature has shown that ERRFI1 is implicated in many diseases, including diabetes, cardiovascular diseases, endotoxemia and cancer (Xu et al. 2006). A previous study revealed that ERRFI1 plays a crucial role in IR-induced pathological events, such as myocardial ischemia (Xu et al. 2006). Here, we provide clear evidence that hepatic ERRFI1 was upregulated in response to IR challenge, and that hepatocyte-specific knockout of ERRFI1 produced beneficial effects against hepatic IR by inhibiting apoptosis and ferroptosis. It has been demonstrated that deletion of ERRFI1 results in decreased apoptosis of epithelial cells during mammary ductal morphogenesis (Hopkins et al. 2012). Consistently, we observed that ERRFI1-deficient mice exhibited less apoptosis of hepatocytes upon IR injury. Notably, our findings first revealed the regulatory effect of ERRFI1 on hepatocyte ferroptosis, and this result might broaden our understanding of the crosstalk between ERRFI1 and cell death.

Apoptosis can be divided into two main categories, internal and external pathways, depending on whether the triggering event is intrinsic or extrinsic to the cell. TNF-α (a major member of the death receptor ligand) and caspase-8 activation have been reported to drive the exogenous apoptotic pathway during hepatic IR injury (Kim et al. 2013). The mitochondria-mediated intrinsic apoptosis pathway occurs when mitochondria are stimulated by ROS and other stimuli, causing a shift in mitochondrial permeability and the release of cytochrome c into the cytoplasm, which ultimately leads to the activation of caspase-3 (Hirakawa et al. 2003). In this study, Bcl-2, Bax, and cleaved caspase-3 were selected to reflect the level of mitochondria-mediated intrinsic apoptosis. Bcl-2 protein is an antiapoptotic member of the Bcl-2 family that maintains mitochondrial membrane stability, whereas Bax protein is a proapoptotic member that destabilizes the mitochondrial membrane (Ouyang et al. 2012). Although increased apoptosis was observed in livers of IR mouse model and OGD/R-induced hepatocytes, ERRFI1 deficiency resulted in weak activation of the intrinsic apoptosis pathway, as evidenced by reduced levels of Bax and cleaved caspase-3, and increased Bcl-2 level. Consistently, Chen et al. suggested that knockdown of ERRFI1 impeded the extent of β-cell apoptosis under stressed conditions in the form of caspase 3 inactivation (Chen et al. 2013).

The process of ferroptosis consists of iron accumulation and lipid peroxidation. Recently, there has been increasing evidence that ferroptosis plays a role in the pathogenesis of hepatic IR (Guo et al. 2022; Wu et al. 2022). In the current study, we demonstrated ERRFI1 deficiency resulted in decreased Fe^2+^ content in livers of IR mice and OGD/R-treated hepatocytes, implying that ERRFI1-triggered iron overload induced ferroptosis during hepatic IR injury. Lipid peroxidation is modulated by multiple crucial factors, such as acyl-CoA synthase long chain 4 (ACSL4), solute carrier family 7 member 11 (SLC7A11), and glutathione peroxidase 4 (GPX4). ACSL4 is one of the essential enzymes in the lipid peroxidation pathway and its high expression facilitates cellular ferroptosis execution (Doll et al. 2017). SLC7A11 transports extracellular cystine into the cell (Wu et al. 2022), which is subsequently converted to glutathione (GSH). GPX4 converts GSH to oxidised glutathione (GSSG) and catalyzes the reduction of lipid hydroperoxides, thereby blocking the ferroptotic cascade (Koppula et al. 2021). Our study proved that ferroptosis suppressors SLC7A11 and GPX4 were reduced while the positive regulator ACSL4 was increased in livers of IR mice and OGD/R-treated hepatocytes. In particular, all of these changes were cancelled by ERRFI1 deficiency, suggesting that ERRFI1 inhibited lipid peroxidation during hepatic IR injury. Therefore, our findings implied that ERRFI1 exacerbated IR-induced hepatic damage by inducing ferroptosis in hepatocytes.

GRB2 is an adaptor protein that couples activated receptor tyrosine kinases to associated downstream molecules (Lowenstein et al. 1992; Skolnik et al. 1993). Previous studies have investigated the involvement of GRB2 in hepatic diseases (Shan et al. 2013; Kondo et al. 2008; Ge et al. 2015). Aligning with the results of a prior study in which GRB2 contributes to palmitic acid-induced hepatocyte apoptosis (Shan et al. 2013), we illuminated that GRB2 acted as a pro-apoptotic modulator of hepatocytes under OGD/R conditions. In addition, we identified an essential role for GRB2 in the induction of ferroptosis of OGD/R-treated hepatocytes. More importantly, the current study provided evidence that the interaction with GRB2 is essential for ERRFI1-mediated cell death in hepatic IR injury. On the basis of our findings, we summarized the mechanisms by which the ERRFI1/GRB2 axis contributed to hepatic IR injury as follows: first, ERRFI1 interacted with GRB2. Second, this interaction facilitated protein stability of GRB2 by hindering proteasomal degradation. Finally, stabilized GRB2 modulated some signal transduction pathways, thus promoting apoptosis and ferroptosis of hepatocyte following hepatic IR. However, we still lack a clear understanding of how ERRFI1/GRB2 axis regulate cell death in hepatic IR injury. The synergistic interaction between ferroptosis and apoptosis can be regulated by endoplasmic reticulum (ER) stress-mediated pathways, which are regarded as a contributor to hepatic IR injury (Peralta and Brenner 2011; Zhang et al. 2022). Considering ERRFI1 exacerbates ER stress (Chen et al. 2013), we speculated that ERRFI1/GRB2 axis may regulate ER stress-mediated pathways and thus enhance the synergistic dual cell death modes during hepatic IR. Further studies are warranted to determine which ER stress-mediated pathway is required for ERRFI1/GRB2 axis-modulated ferroptosis and apoptosis.

In summary, we uncovered ERRFI1 as a prominent initiator of hepatic IR injury. ERRFI1 directly interacts with GRB2 and maintains the protein stability of GRB2 via the ubiquitin-proteasome pathway, leading to hepatocyte apoptosis and ferroptosis, thereby contributing to hepatic IR injury. Our findings provide a theoretical basis for proposing potential approaches to prevent or treat hepatic IR injury.

### Electronic supplementary material

Below is the link to the electronic supplementary material.

Supplementary Material 1: Oil-Red-O staining of liver tissues from WT mice and ERRFI1-HKO mice.

## Data Availability

The dataset supporting the conclusions of this article is available in the Biogrid website (https://thebiogrid.org/).

## Abbreviations

IR: Ischemia and reperfusion
OGD/R: Oxygen-glucose deprivation/reoxygenation
ERRFI1: ERBB receptor feedback inhibitor 1
ERRFI1-HKO: Hepatocyte-specific ERRFI1 knockout
WT: Wild-type
ALT: Alanine aminotransferase
AST: Aspartate aminotransferase
GSH: Glutathione
MDA: Malondialdehyde
H&E: Hematoxylin and eosin
Fe^2+^: Ferrous iron
BSA: Bovine serum albumin
DAB: 3,3’-diaminobenzidine
FBS: Fetal bovine serum
CHX: Cycloheximide
ACSL4: Acyl-CoA synthase long chain 4
SLC7A11: Solute carrier family 7 member 11
GPX4: Glutathione peroxidase 4
GRB2: Growth factor receptor-bound protein 2
