# Supplementary figures and images for: ERRFI1 exacerbates hepatic ischemia reperfusion injury by promoting hepatocyte apoptosis and ferroptosis in a GRB2-dependent manner
Source: Mol Med. 2024 Jun 11;30:82. doi: 10.1186/s10020-024-00837-4 (PMC11167874; doi:10.1186/s10020-024-00837-4)

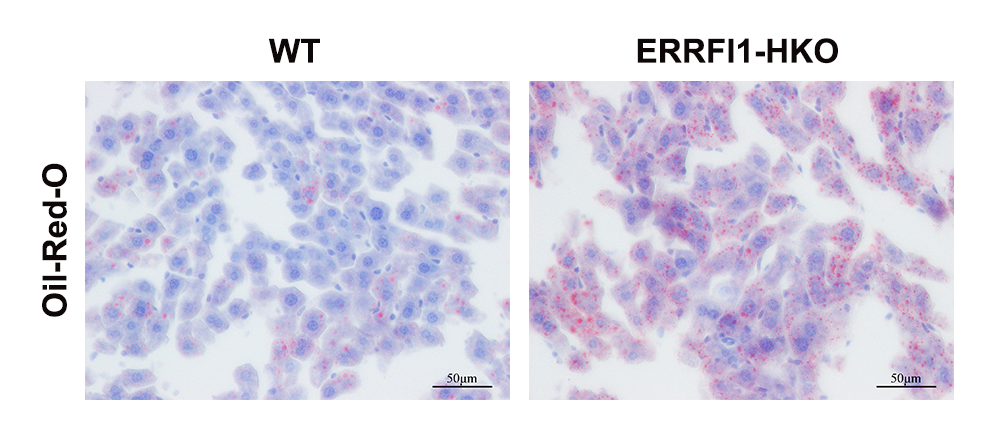

Supplement: Supplementary file 1 — Supplementary Material 1: Oil-Red-O staining of liver tissues from WT mice and ERRFI1-HKO mice. [file 10020_2024_837_MOESM1_ESM.tif]
